# Metabolic Changes in *Avena sativa* Crowns Recovering from Freezing

**DOI:** 10.1371/journal.pone.0093085

**Published:** 2014-03-27

**Authors:** Cynthia A. Henson, Stanley H. Duke, David P. Livingston

**Affiliations:** 1 United States Department of Agriculture-Agricultural Research Service, Cereal Crops Research Unit, Madison, Wisconsin, United States of America; 2 Department of Agronomy, University of Wisconsin, Madison, Wisconsin, United States of America; 3 United States Department of Agriculture-Agricultural Research Service and Department of Crop Science, North Carolina State University, Raleigh, North Carolina, United States of America; University of Delhi South Campus, India

## Abstract

Extensive research has been conducted on cold acclimation and freezing tolerance of fall-sown cereal plants due to their economic importance; however, little has been reported on the biochemical changes occurring over time after the freezing conditions are replaced by conditions favorable for recovery and growth such as would occur during spring. In this study, GC-MS was used to detect metabolic changes in the overwintering crown tissue of oat (*Avena sativa* L.) during a fourteen day time-course after freezing. Metabolomic analysis revealed increases in most amino acids, particularly proline, 5-oxoproline and arginine, which increased greatly in crowns that were frozen compared to controls and correlated very significantly with days after freezing. In contrast, sugar and sugar related metabolites were little changed by freezing, except sucrose and fructose which decreased dramatically. In frozen tissue all TCA cycle metabolites, especially citrate and malate, decreased in relation to unfrozen tissue. Alterations in some amino acid pools after freezing were similar to those observed in cold acclimation whereas most changes in sugar pools after freezing were not. These similarities and differences suggest that there are common as well as unique genetic mechanisms between these two environmental conditions that are crucial to the winter survival of plants.

## Introduction

Winter cereals such as rye, wheat, barley and oat typically have better yields than their spring counterparts because they can be harvested earlier in the growing season before the normally hot and dry conditions of summer are encountered. The major limitation to growing a fall sown crop is a lack of resistance to adverse conditions over winter. Favorable cold temperatures above freezing during the fall elicit changes in gene expression and in various physiological and biochemical processes resulting in the development of cold acclimation, which has been reviewed extensively [1]–[5]. In addition to cold acclimation at temperatures above freezing, acclimation at below freezing temperatures confers hardiness beyond that achieved during cold acclimation. This subfreezing acclimation has been called “second phase hardening” [6]–[8] and “subzero acclimation” [9]–[12].

The involvement of a large number of genes in both cold and in subzero acclimation has been documented in many grass and legume plant species [e.g. 13–18] and attempts to improve winter hardiness frequently exploit DNA markers of these genetic differences. Wooten et al. [19] mapped 23 restriction fragment length polymorphic markers in oats. The markers were identified by Santos et al. [20] based on their proximity to a chromosome translocation and association with 21 simple sequence repeats (SSR) developed from oat genomic libraries [21]; [22]. SSR’s associated with freezing tolerance are routinely identified in the Uniform Oat Winter Hardiness Nursery (http://www.ars.usda.gov/Main/docs.htm?docid=8419 *)* and are used by plant breeders as an aid in selection of freezing tolerant germplasm. In addition to oats, QTLs associated with winter hardiness in cereals have been identified in diploid wheat (*Triticum monococcum*) [23], bread wheat (*T. aestivum* L.) [24]–[27], and barley (*Hordeum vulgare* L.) [15]; [28]; [29]. Almost all these QTLs in both grasses and in legumes [13]; [30] are also linked to QTLs for other winter hardiness component traits, such as the vernalization response to flowering.

Acclimation to both cold temperatures above freezing and to temperatures below freezing are two critical phases of winter hardiness with a third being recovery upon alleviation of the temperature stress (e.g. return to more optimal growth temperatures). Histological analyses of cold acclimated oat crowns, which are the overwintering tissues, that have undergone freezing then subsequently thawed, such as would occur during the production of fall sown cereals, indicated dramatic differences between frozen and unfrozen cold acclimated crowns [31]. Similar differences have been reported in wheat and barley [32], and other grass species that have been frozen and thawed [33]; [34]. More recently, histological analysis and 3D reconstruction of crown tissues of wheat, barley, oat and rye during a period following freezing revealed a structural change that was unique to oats [31]. During the 14 day post-freezing period oat crowns developed a distinct region of cells appearing as a ring of lignified tissue with nonviable cells, as determined by vital staining with tetrazolium, being within the ring and viable cells outside of the ring.

Significant changes in metabolism are known to occur during both cold and subzero acclimation [4]; [35]; however, with the exception of a study on carbohydrate changes in oat crowns that had been frozen [8], little research has been reported on metabolic changes during the post-freezing period of recovery. It is possible that many metabolic changes are related to repair mechanisms of the plant and are therefore essential aspects of overall winter hardiness. For example, Palta et al. [36] demonstrated an alteration in the permeability of onion cells during freezing recovery of whole tissues and reported that the use of the popular electrolyte leakage test as a single time point measurement immediately after freezing was an inadequate measure of injury because cell leakage was dramatically lower several days after freezing compared to immediately thereafter. They suggested repair of cellular membranes occurred during recovery that resulted in reduced stress-induced permeability and showed that electrolyte leakage (primarily K^+^) from cells may be totally reversible depending on the degree of freezing injury [36]; [37]. In contrast, leakage of larger cellular metabolites, such as vacuolar fructans, and organellar macromolecules, such as malate dehydrogenase, glutamate dehydrogenase, glutamate oxaloacetate transaminase, etc. or other proteins which have high molecular weights would indicate massive cellular rupture of at least some cells in tissues being tested, regardless of the cause of cellular injury [e.g. 38–43].

The work reported here was to determine if the recovery phase is metabolically similar to or distinct from those metabolic changes that occur during cold and freezing acclimation that collectively impart winter hardiness. To do this, metabolic changes during the post-freezing period of oat crowns that had been cold acclimated were analyzed via GC-MS.

## Materials and Methods

### Plant growth

Seeds of *Avena sativa* cv. Wintok were planted in Scotts Metromix 220 (Scotts-Sierra Horticultural Products Co., Marysville, OH) in plastic tubes (2.5 cm diameter × 16 cm high) with holes at the bottom for drainage and suspended in racks. Plants were treated twice weekly with a modified Hoagland’s nutrient solution (8) and flushed three times weekly with water. Plants were grown for 5 weeks at a day/night temperature regime of 13 to 10°C with a 12 h photoperiod in a growth chamber with 240 μmol m^−2^s^−1^ PAR (80% cool white fluorescent and 20% incandescent). These were nonacclimated (NA) plants.

### Cold acclimation

Five weeks after planting, plants were transferred to a chamber at 3°C with a 12 h photoperiod at 310 μmol m^−2^s^−1^ for 3 weeks. This period constituted cold acclimation (CA). Plants at this stage had three visible tillers with one large tiller in the center sometimes splitting into two stems. This larger, central tiller was designated the primary tiller.

### Freezing tests

The soil surface of racks containing CA plants was sprinkled with ice shavings to promote freezing and prevent supercooling. Racks with plants in alternating rows, to allow better heat distribution, were completely covered with a plastic bag to minimize desiccation during freezing. Plastic bags were loosely sealed and placed into a freezer at –3°C. The soil temperature was monitored with thermocouples. After the soil was completely frozen, which took approximately 6 h, the freezer temperature was lowered 1°C h^−1^ to –12°C. The freezers were held at –12°C for 3 h and then gradually warmed to 3°C at a rate of 2°C h^−1^. When the soil temperature reached 0°C racks were removed and allowed to completely thaw under CA (3°C with light) conditions. After thawing they were moved to NA (13°C) growing conditions. Plants were removed and primary tillers excised at 0, 1, 3, 7 and 14 d for metabolomics analysis. Only the primary tillers were used for both histological and metabolomic analysis and are referred to as the crown.

### Tetrazolium analysis

Crowns at day 0,1,3,7 and 14 were cut longitudinally with a razor and incubated with 0.5% 2,3,5-triphenyltetrazolium chloride in 50 mM HEPES (pH 7.3) for 24h at 21°C. Unfrozen controls were incubated in an identical solution. The cut surfaces of the crowns were photographed under a dissecting microscope at 12X with surface lighting. The red color was quantified using SigmaScan Pro (version 5.0, Systat Software, Inc., San Jose, CA).

### Safranin analysis

Staining with safranin and quantitative analysis of the staining response was conducted on crown sections as described by Livingston et al. [31].

### Metabolite extraction and analysis

Primary tillers from crowns of five plants per time point were ground in 1.5 ml Eppendorf tubes in an amalgamator for 10 s then heated at 90°C for 15 min. Ground samples were frozen at –80°C until all samples were collected (3 weeks). Ground tissues were extracted in 100% methanol (HPLC grade) by shaking at 150 rpm at 70°C for 90 min, followed by addition of Milli-Q water (18Ω) and additional shaking for 10 min at 70°C at ratios of 100 mg tissue: 100 μl methanol: 33 μl H_2_O, then centrifuged at 18,500 x *g* for 10 min. Supernatants were divided into six equal portions and lyophilized at –80°C for a minimum of 5 h.

Samples were loaded onto a Shimadzu AOC-5000 Auto Injector for automatic derivatization with two protocols similar to that of Roessner et al. [44]. The only difference in the two derivatization protocols was that one was initiated by the addition of 10 μl methoxyamine HCL (#33045U, Sigma-Aldrich) in pyridine (200 mg mL^−1^; #270407U, Sigma-Aldrich) with internal standards and the second derivatization procedure was initiated by addition of only pyridine with internal standards. Internal standards were 1-tridecene, 1-undecanol, 1-heptadecanol and methyl tricosanoate at 160 ng/μl. The remaining steps of derivatization were the same for all samples. After initiation of derivatization, samples were shaken at 70°C for 90 min, then 10 μl of BSTFA (*N,O*-bis[trimethylsilyl]trifluoroacetamide) with TCMS (trimethylchlorosilane, 99:1, #33155U, Sigma-Aldrich) was added, followed by shaking for 90 min at 70°C.

Each 3 μl sample was injected onto the Shimadzu GC/MS-QP5050A by the auto injector immediately upon completion of derivatization. The GC injector was in the split/splitless mode (44:1) at 300°C and the interface temperature was 290°C. Metabolites were separated on a Zebron ZB-50 capillary column (30 mm×0.25 mm ID; 0.25 μm film thickness; Phenomenex, Torrance, CA) with helium carrier gas at a flow rate of 1.5 ml min^−1^. For samples derivatized with the first step containing methoxyamine HCL, the oven temperature was programmed at 70°C for 5 min, followed by a 5°C min^−1^ ramp to 310°C, and held at 310°C for a total run time of 58 min. The system was then equilibrated for 90 min at 70°C prior to injection of the next sample. MS detector voltage was maintained between 1.2 kV to 1.65 kV to allow compounds of varying concentrations to remain on scale. For samples derivatized with the first step containing pyridine only, the voltage detector varied from 1.2 kV to 1.7 kV and the oven temperature program was 70°C for 5 min, followed by a 5°C min^−1^ ramp to 260°C. At this time the detector was shut off and the oven temperature ramped at 20°C min^−1^ to 310°C before being cooled to 70°C for equilibration. All mass spectra were recorded in the electron impact mode at 2000 scans s^−1^ with *m/z* scanning range of 28 to 750 amu.

Peak identification, done after deconvolution via AMDIS, was through use of the Wiley Registry of Mass Spectral Data - 7^th^ edition, the SZTERP MS library of flavors, fragrances and essential oils, NIST 05 and our in-house library from authentic standards. Peaks were identified based on spectral data and retention index from the in-house library where information was present; otherwise, putative identification is based on spectral data alone.

Statistical evaluations of metabolites used the total ion count of each peak expressed as a percent of the sum of all peaks in a sample. SAS V 2 (SAS Institute, Cary, NC) was used for analysis of variance with a t-test comparing group means. The Pooled test was used where the equality of variances *P*r>F was greater than 0.05 and the Statterthwaite test used where the *P*r<F was less than 0.05. The equality of variances in t-tests addressed non-normality issues. The Benjamini-Hochberg false discovery rate adjusted *P*-values were used to determine if metabolite abundance in frozen crowns was significantly different from that in unfrozen crowns. No correction was made for t-tests of differences in safranin staining between frozen and control crowns as tissues stained with safranin were generated and analyzed in a separate but parallel experiment resulting in only 5 t- tests being calculated for those crowns. Regression analysis and correlations between variations in metabolite ion counts over time and the extent of safranin staining, as described by Livingston et al. [31] were analyzed using PROC CORR. Principal Component Analysis (PCA) was conducted using Pirouette (Woodinville, WA) in the autoscale function, with cross-validation by the 1-step method, and outliers were removed. Loading values for the first five principal components are in Table S1.

## Results and Discussion

### Identification of live cells within the crown

To determine the percentage of live cells in longitudinal sections of crowns staining with 2,3,5- triphenyl tetrazolium chloride was conducted. This stain is routinely used to test the viability of seed that has been stored [45] and has also been used as an indicator of vegetative cells which survived freezing [46]; [47]. Tetrazolium is reduced by electrons from the electron transport system of the mitochondria [48] forming a red-colored derivative, formazan, allowing the visual distinction of live (red) from dead (white) cells.

**Figure 1:**
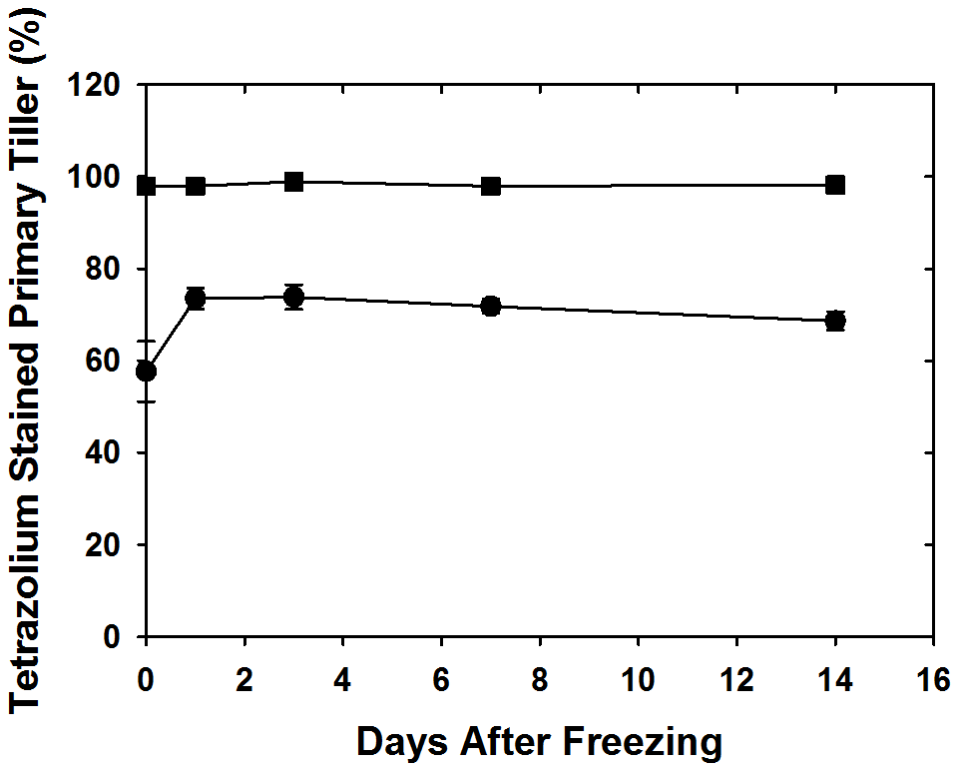
Changes in percent tetrazolium stained area of frozen (circles) and control (squares) crowns (primary tillers) of cold acclimated oats during 0 to 14 days of growth after imposition of freezing stress.

Tetrazolium reduction in crowns immediately after freezing (day 0) indicated that 57% of cells were able to reduce tetrazolium (Figure 1). There was a significant increase in the percentage of red cells between day 0 and day one (57% to 73%), suggesting recovery of these plants. After day 1 the percentage of live cells only slightly changed up to day 14 when 68% of cells were capable of reducing tetrazolium.

### Metabolomics

Although considerable literature exists on metabolic changes in leaves of plants cold acclimated at temperatures above [49]–[54] or below freezing [55], very little literature is available that addresses changes in metabolism in plants regrowing at optimal conditions after having been frozen. Mazzucotelli et al. [55] documented changes in amino acids and related metabolites in wheat and barley leaves 48 h after being frozen for 16 h at –3°C. However, it is unlikely that full effects of the freeze had developed 48 h after freezing. Additionally, leaves of winter barley and wheat are not the overwintering tissues. To our knowledge, the only report of metabolic changes occurring in crowns, the actual overwintering tissues of winter cereals, during a period of recovery after freezing is that of Livingston [8], which documented changes in fructans and related mono- and disaccharides in oat crowns. Although the two biological systems (leaves vs. crowns and cold acclimation vs. post-freeze response under nonacclimating conditions) are composed of different tissues at different developmental stages, their metabolic responses to low temperature appear to have similarities, especially in the case of proline and arginine, as well as distinct differences, most notably in sugar metabolism.

#### Amino acids

Statistically significant differences in abundances of 13 amino acids that occur naturally in proteins (arginine, asparagine, aspartate, glutamate, glycine, histidine, isoleucine, leucine, proline, serine, threonine, tryptophan, valine) and two metabolites resulting directly from amino acid catabolism or interconversion (5-oxoproline, piperidinecarboxylate) between frozen and nonfrozen oat crowns were observed on two or more days after freezing (DAF) (Table 1; Figure S1). Levels of aspartate and glutamate were lower in crowns that had been frozen than in nonfrozen crowns on 1 to14 DAF. In contrast, on days where significant differences existed, levels of the remaining 11 amino acids and two related metabolites were higher in crowns that had been frozen than in nonfrozen crowns, with two exceptions. Threonine and 5-oxoproline levels were initially higher in the controls, yet subsequently increased in frozen crowns above the levels present in control crowns.

**Table 1:** Statistical significance of the differences in amino acid related metabolite levels and safranin staining between frozen and nonfrozen oat crowns during days 0 to 14 after freezing.

	Day 0	Day 1	Day 3	Day 7	Day 14	*r^2^*Days	Slope	*r* ^2^Safranin
Safranin Staining and Metabolite								
Safranin Staining	ns^a^	ns	ns	*↑ b ^,^ c	*↑	0.98****	0.82****	
Alanine	ns	ns	ns	ns	ns	0.66****	–0.18****	0.61****
Arginine	ns	ns	ns	***↑	***↑	0.94****	0.11****	0.92****
Asparagine	**↓	ns	**↑↑	**↑↑↑	***↑↑↑↑	0.82****	0.21****	0.80****
Aspartate	**↓	*↓	**↓	*↓	*↓	0.64****	0.16****	0.47***
Glutamate	**↓	**↓	*↓	**↓	**↓↓	0.44***	–0.26****	0.47***
Glycine	ns	ns	*↑↑	*↑↑	ns	ns	ns	ns
Histidine	nd^d^	nd	ns	**↑	**↑	0.84****	0.07****	0.80****
Isoleucine	ns	ns	***↑↑↑	***↑↑↑	**↑↑↑	0.45***	0.16***	0.51****
Leucine	ns	ns	ns	***↑↑	***↑↑	0.57****	0.07***	0.66****
Lysine	ns	ns	ns	ns	***↑↑↑	0.85****	0.05****	0.80****
Phenylalanine	ns	ns	ns	ns	ns	ns	ns	ns
Proline	**↓	*↑	***↑↑↑	***↑↑↑	***↑↑↑	0.51****	0.65****	0.49****
Serine	**↓	ns	**↑	**↑	**↑	ns	ns	ns
Threonine	***↓	ns	**↑	***↑	**↑	0.45***	0.17***	0.47***
Tryptophan	nd	ns	ns	*↑	**↑	0.81****	0.06****	0.77****
Valine	*↓	*↑	***↑↑	***↑↑	***↑↑	0.60****	0.30****	0.63****
γ-Aminobutyrate	ns	ns	ns	ns	ns	0.23*	–0.14*	0.18*
β-Alanine	nd	ns	nd	ns	ns	ns	ns	ns
5-Oxoproline	***↓↓	ns	***↑↑	***↑↑	**↑↑↑	0.74****	0.80****	0.72****
Piperidinecarboxylate	ns	ns	**↑↑↑↑	*↑↑↑	*↑↑↑↑	0.61****	0.12****	0.59****

a ns = not significant.

b *, **, *** and **** Denotes statistical significance at the *P*≤0.05, 0.01, 0.001 and 0.0001 levels, respectively, using a two-tailed t-test with FDR correction via Benjamini-Hochberg.

c Denotes the metabolite was higher (↑) or lower (↓) in the frozen samples. One, two, three or four arrows indicate metabolite changes were ≤2-, >2≤5-, >5≤10, >10-fold, respectively.

d nd = no metabolite detected.

“*r* ^2^Days” values are the coefficients of determination (correlation coefficients squared) of the changes in safranin staining or in metabolite levels during days 1 to 14 of growth after freezing. “*r* ^2^Safranin” values are the coefficients of determination of the changes in metabolite levels in frozen crowns with the development of safranin staining tissue during days 1 to 14 of growth after freezing.

Free proline levels increased significantly in the frozen crowns relative to control crowns at 1 d, 3 d, 7 d and 14 d of regrowth and the coefficients of determination, and thus the correlation coefficients, were highly significant with DAF (Table 1). The positive slope of proline accumulation after freezing was steeper than for any other metabolite with the exception of 5-oxoproline (Tables 1–3). Proline accumulation in plants has long been known to be associated with a number of abiotic and biotic stresses [56], including both low and high temperature [e.g. 54–62]. Also, a study of temperature shock (heat and cold) with *Arabidopsis thaliana* indicated that proline is increased by cold shock but not with heat shock [63]. Free proline protects enzymes from denaturation [64]–[66] and stabilizes membranes [67] and polyribosomes [68]. Proline incorporation into proteins increases resistance to thermal denaturation. Proline residues are important for enzyme thermostability because increases in the frequency of proline in β-turns and in the total number of hydrophobic residues enhance protein stability [69]; [70]. The insertion of just one proline residue into barley α-glucosidase increases thermostability considerably [71]. Even in soybean, a chilling sensitive species, grown at low temperature, proline content increased by 350% in the axes of germinating seeds [72].

**Table 2:** Statistical significance of the differences in sugar related metabolite levels between frozen and nonfrozen oat crowns during days 0 to 14 after freezing.

	Day 0	Day 1	Day 3	Day 7	Day 14	*r* ^2^Days	Slope	*r* ^2^Safranin
Safranin Staining and Metabolite								
Safranin Staining	ns^a^	ns	ns	*↑^b^^,^ c	*↑	0.98****	0.82****	
Arabinoate	ns	ns	ns	ns	ns	ns	ns	ns
Arabinose	ns	***↓↓↓	ns	*↑↑	ns	0.29**	0.01**	0.35**
Erythrose	ns	ns	ns	ns	ns	ns	ns	ns
Erythritol	*↑	ns	*↑	ns	ns	ns	ns	0.20*
Fructose	***↑	**↑	*↑	ns	ns	0.27**	–0.38**	0.29**
Galactose	ns	ns	ns	ns	**↑↑↑	ns	ns	ns
Glucose	ns	ns	ns	ns	ns	ns	ns	ns
Glucuronate	ns	ns	ns	ns	ns	ns	ns	ns
Gulose	**↓↓	ns	ns	ns	***↓↓	0.32**	–0.01**	0.30**
2-keto-gluconate	ns	ns	ns	ns	ns	ns	ns	ns
4-keto-glucose	ns	ns	ns	ns	ns	ns	ns	ns
Mannose	ns	ns	ns	ns	ns	ns	ns	ns
Melibiose	ns	ns	ns	ns	ns	0.23*	–0.03*	0.22*
*Myo*-inositol	*↓	ns	ns	ns	**↓	0.27**	–0.03**	0.24*
Palatinose	ns	ns	ns	ns	ns	ns	ns	ns
Sucrose	*↓	ns	**↓	ns	**↓	0.51****	–0.79****	0.48****
Sorbitol	ns	ns	ns	ns	ns	0.41***	0.04***	0.42***
Turanose	*↓↓	ns	ns	ns	ns	0.21*	0.17*	0.21*
Xylulose	ns	*↑	ns	ns	ns	0.23*	–0.03*	0.24*

a ns = not significant.

b *, **, *** and **** Denotes statistical significance at the *P*≤0.05, 0.01, 0.001 and 0.0001 levels, respectively, using a two-tailed t-test with FDR correction via Benjamini-Hochberg.

c Denotes the metabolite was higher (↑) or lower (↓) in the frozen samples. One, two, three or four arrows indicate metabolite changes were ≤2-, >2≤5-, >5≤10, >10-fold, respectively.

“*r* ^2^Days” values are the coefficients of determination (correlation coefficients squared) of the changes in safranin staining or in metabolite levels during days 1 to 14 of growth after freezing. “r*^2^*Safranin” values are the coefficients of determination of the changes in metabolite levels in frozen crowns with the development of safranin staining tissue during days 1 to 14 after freezing.

**Table 3:** Statistical significance of the differences in metabolite levels between frozen and nonfrozen oat crowns during days 0 to 14 after freezing.

	Day 0	Day 1	Day 3	Day 7	Day 14	*r* ^2^Days	Slope	*r* ^2^Safranin
Safranin Staining and Metabolite								
Safranin Staining	ns^a^	ns	ns	*↑ b ^,^ c	*↑	0.98****	0.82****	
Acetate	ns	ns	ns	ns	ns	ns	ns	ns
Cis-Aconitate	ns	ns	ns	ns	**↓↓	0.44***	–0.04***	0.44***
Citrate	ns	ns	ns	**↓	***↓↓	0.76****	–0.53****	0.78****
Fumarate	*↑↑↑	ns	ns	*↓	ns	ns	–0.13**	ns
Isocitrate	ns	ns	ns	ns	ns	0.29**	–0.001****	0.30**
Malate	*↑	ns	***↓	**↓↓	***↓↓	0.80****	–1.39****	0.80****
Succinate	***↑	*↓	***↓↓	*↓	**↓↓	030**	–0.13****	0.29**
Butanoate	ns	ns	*↑	*↑↑	**↑↑↑	0.51****	0.005****	0.52****
Glycolate	ns	ns	ns	ns	ns	ns	ns	ns
Lactate	**↑↑	ns	*↑↑	ns	*↑↑	0.40***	–0.12***	0.38***
Glycerol	**↑↑	ns	ns	*↓	ns	0.53****	–0.10****	0.51****
Linoleic acid	ns	ns	ns	ns	ns	ns	ns	0.16*
Linolenic acid	ns	nd^d^	ns	nd	nd	ns	ns	ns
Palmitic acid	***↑↑	ns	ns	*↑↑	ns	ns	ns	ns
Stearic acid	ns	*↑	ns	ns	ns	0.32**	–0.01**	0.31**
2,5-furandione	ns	ns	nd	nd	nd	ns	ns	ns
Rythronic acid	ns	ns	ns	ns	**↑	0.64****	0.01****	0.63****
Bis-propyl-phosphoric acid	ns	ns	ns	ns	ns	ns	ns	ns
Phosphoric acid	ns	**↓	ns	*↓	*↓	0.63****	0.32****	0.63****
Cinnamic acid	*↑	ns	ns	*↓↓	*↓↓	0.49****	–0.01****	0.48****
Dihydroxy-coumarin	ns	ns	ns	ns	ns	ns	ns	ns
Vanillic acid	**↑	nd	nd	ns	ns	0.17*	–0.0004*	ns

a ns = not significant.

b *, **, *** and **** Denotes statistical significance at the *P*≤0.05, 0.01, 0.001 and 0.0001 levels, respectively, using a two-tailed t-test with FDR correction via Benjamini-Hochberg.

c Denotes the metabolite was higher (↑) or lower (↓) in the frozen samples. One, two, three or four arrows indicate metabolite changes were ≤2-, >2≤5-, >5≤10, >10-fold, respectively.

d nd = no metabolite detected.

“*r* ^2^Days” values are the coefficients of determination (correlation coefficients squared) of the changes in safranin staining or in metabolite levels during days 1 to 14 of growth after freezing. “*r* ^2^Safranin” values are the coefficients of determination of the changes in metabolite levels in frozen crowns with the development of safranin staining tissue during days 1 to 14 of growth after freezing.

Significant increases in arginine levels in crowns that were frozen relative to control crowns occurred at 7 d and 14 d (*P*≤0.001) and coefficients of determinations were very significantly associated with DAF (*P*≤0.0001) (Table 1). The coefficients of determination of arginine versus DAF were the highest observed (Table 1), suggesting a very close relationship between response to freezing, free arginine pools, and the formation of the spherical structure in the interior of frozen tillers [31]. A study of metabolic changes in populations of *Arabidopsis lyrata* ssp. *petraea* from Iceland, Sweden, and Wales, locations selected for their latitudinal gradient, found that, at low temperature (5 °C), plants from Iceland, the most northern latitude and with phenotypes that were the most cold hardy, arginine content increased very significantly (*P*≤0.001) over plants grown at 20 °C [52]. This was not observed in phenotypes from the more southern latitudes of Sweden and Wales. Thus, increases in free arginine appear to be related to both cold acclimation and the response to freezing in higher plants. Also, the study of temperature shock with *Arabidopsis thaliana* indicated that arginine is increased by cold shock but not with heat shock [63]. Arginine has been demonstrated to strongly protect plant protoplasts from lysis due to osmotic shock [73]. In that study, an increase in polyamines metabolically related to arginine and lysine (cadaverine, spermidine and putrescine), was suggested as a possible mode of action of lysis protection from osmotic shock. In barley leaves, levels of proteins with arginine rich basic domains increase dramatically with cold acclimation [74].

In addition to proline and arginine, the glutamate family of amino acids, which originates from α-ketoglutarate, also includes glutamate, glutamine and γ-aminobutyrate (GABA). Changes in glutamate levels (slope  =  –0.26; *r* ^2^Day  =  0.44) were significantly reduced in frozen crowns at 1 d to14 d (Table 1). There were no significant differences in GABA levels between crowns that had been frozen and unfrozen controls during regrowth, although GABA levels decreased somewhat over time in both frozen crowns (slope  =  –0.14; *r* ^2^Day  =  0.23) and controls. Glutamine was not detected in our study of oat crowns recovering from freezing. The aforementioned study of intraspecific variation in metabolic changes in leaves of three populations of *Arabidopsis lyrata* ssp. *petraea* [52] revealed that cold acclimation at 5°C induced significant increases in glutamate, glutamine and GABA in two populations. They also showed that the largest significant increase in amino acids in cold acclimated plants occurred in glutamine. The CBF cold response pathway reportedly includes significant increases in glutamate and glutamine in cold acclimated *Arabidopsis* when *CBF* expression was up regulated [49]. Results of Skinner et al. [75] demonstrate that expression of some members of the *CBF* gene family play a role in the development of winter hardiness in some barley germplasm, although that study did not address changes in the metabolome [75]. Glutamate and GABA increased in wheat shoots during acclimation at 2°C for 21 d [54]. Mazzucotelli et al. [55] reported that levels of GABA increased and glutamate decreased in leaves of cold acclimated barley that were stressed at –3°C and that GABA further increased and glutamate further decreased as a function of an additional exposure to –8°C. They also found that –3°C treated barley seedlings allowed a short period of recovery at 22°C, resulted in continued decrease in glutamate and an approximately 20-fold decrease in GABA. Although the GABA shunt pathway of glutamate degradation is frequently implicated as involved in low temperature responses of different tissues from several plant species undergoing a variety of different cold acclimation regimes, no single picture has clearly emerged. The results of Mazzucotelli et al. [55], although on subzero acclimated leaves of barley, combined with those we present here, when compared to the aforementioned studies of cold acclimated wheat shoots and *Arabidopsis* leaves, suggest that GABA and glutamate responses to acclimation and recovery from freezing are indeed different.

Aspartate levels in frozen crowns decreased relative to levels in nonfrozen controls significantly at 1 d, 3 d, 7 d and 14 d (*P*≤0.05) after freezing (Table 1). The coefficient of determination of changes in aspartate levels of frozen crowns with DAF was very highly significant with a slight, positive and highly significant slope (*r* ^2^Days  =  0.64; *P*≤0.0001), indicating that aspartate increased in both frozen and nonfrozen tissues. In the study with *Arabidopsis lyrata* ssp. *petraea* populations from different latitudes [52], under cold acclimating conditions, aspartate increased significantly in plants from Iceland (*P*≤0.001, highest latitude) and Sweden (*P*≤0.05, middle latitude) but not in plants from Wales (lowest latitude). This suggests that survival at low temperature may be somewhat dependent on aspartate accumulation. When plant species that overwinter, such as alfalfa, are exposed to low temperature, very significant increases in enzymes such as glutamate oxaloacetate transaminase (GOT) occur in the tissues that overwinter [76]. In that study GOT activity increased more than 400-fold in the taproots of the most cold hardy cultivar (grown in Canada), when placed under cold acclimating conditions as compared to no increase in GOT activity in the least cold hardy cultivar (grown in Arizona and Mexico). Changes in levels of GOT with cold acclimation could affect levels of aspartate in that GOT can convert glutamate and oxaloacetate to aspartate and α-ketoglutarate via transamination.

After freezing, asparagine significantly increased in frozen crowns compared to control crowns at 3, 7 (*P*≤0.01) and 14 d (*P*≤0.001) (Table 1). Also, the coefficient of determination of changes in asparagine levels with DAF and the slope were highly significant (*P*≤0.0001). In the study with *Arabidopsis lyrata* ssp. *petraea* populations from different latitudes, under cold acclimating conditions asparagine only increased significantly in plants from Iceland (*P*≤0.05, highest latitude) [52]. Our results and those of Davey et al. [52] compared to results from a microarray study with *Arabidopsis* showing the gene for asparagine synthetase was down regulated by cold and other stresses [77] suggest that changes in asparagine levels in response to cold temperature may be independent of transcript abundance. Asparagine synthetase is the primary mechanism for asparagine synthesis in plants [78], and without its activity or with much lowered activity, one would expect to see a decrease in asparagine if the gene for asparagine synthetase is down regulated with cold stress. Alternatively, regulation of asparagine levels in response to cold temperature may occur through changes in asparagine utilization and/or catabolism.

Valine, leucine and isoleucine, the three branched chain amino acids, all increased in abundance after freezing, with *r* ^2^Day values of 0.60, 0.57 and 0.45, respectively (Table 1). Valine and leucine have been shown to increase in leaves of *Arabidopsis* exposed to 8°C for 6 and 72 h relative to 20°C controls [51] and increases in all three branched chain amino acids were documented when exposure was at 4°C [50]. Valine and leucine are synthesized from pyruvate and isoleucine is synthesized from oxaloacetate, neither of which was detected. However, it could be speculated that *in planta* levels of oxaloacetate decreased in crowns recovering from freezing because other TCA cycle intermediates decreased (Table 3). Valine, leucine and isoleucine increased in wheat shoots acclimated at 2°C for 21 d [54]. Recently, leucine has been shown to mediate expression of perhaps as many as several hundred *Arabidopsis* genes in several functional categories including degradation of glutamate, aspartate and proline [79].

Glycine levels in frozen crowns increased significantly at 3 and 7 d of regrowth compared to the control crowns yet the coefficients of determination with DAF were not significant and the slope of changes in glycine levels vs. DAF was not significant (Table 1). In the study with *Arabidopsis lyrata* ssp. *petraea* from Iceland, Sweden, and Wales, low temperature increased glycine concentrations significantly in biotypes from Iceland and Wales, whereas, curiously, those from Sweden were not significantly increased [52]. Many proteins with glycine rich domains, such as the COR/LEA proteins, are produced during cold acclimation [80]. These glycine rich proteins have a stabilizing effect on membranes [81]; [82]. Glycine-rich RNA-binding proteins which are associated with plant cold hardiness [83] could be associated with increases in glycine.

Serine levels in frozen crowns increased significantly above levels in controls at 3 d, 7 d and 14 d although the coefficients of determination with DAF were not significant, nor was the slope of changes in serine levels vs. DAF significant (Table 1). Serine levels have been shown to increase in leaves of *Arabidopsis thaliana* during cold acclimation at above freezing temperatures [50]. Serine levels have also been shown to decrease in one *A. lyrata* ssp. *petraea* population and to have no significant changes in two other populations selected for variation in latitudinal adaptation [52] during cold acclimation at above freezing conditions. Serine levels in wheat shoots acclimated at above freezing temperatures increased relative to controls [54].

At 3 d, 7 d and 14 d threonine concentrations increased significantly (*P*≤0.01) above levels in unfrozen controls and the coefficients of determination with DAF were significant (*P*≤0.001) (Table 1). The slope of the increase was moderate but highly significant (*P*≤0.001). In contrast, in the study with *Arabidopsis lyrata* ssp. *petraea* populations from different latitudes [52], there were no significant increases in threonine concentrations in plants from highest to lowest latitudes. In some prokaryotes, genes involved in threonine are up regulated with cold shock [84].

Histidine significantly increased in frozen crowns at 7 d and 14 d (*P*≤0.01) compared to control crowns (Table 1). The coefficient of determination of changes in histidine levels with DAF was very significant (*P*≤0.0001), although the slope was slight. The association of histidine with DAF was the third best of all metabolites, lower only than arginine and lysine. In the study with *Arabidopsis lyrata* ssp. *petraea* populations from different latitudes [52], histidine increased significantly (*P*≤0.01) only in plants from Iceland under cold acclimating conditions. Although membrane associated histidine kinases have been determined to be possible cold sensors [85], the possibility that increases in free histidine are associated with increases in histidine kinases has not been tested.

At 14 DAF lysine concentrations increased very significantly (*P*≤0.001) in frozen crowns as compared to the nonfrozen crowns. The coefficient of determination of changes of lysine levels with DAF was highly significant and, although the slope of the increase was fairly low, it also was highly significant (*P*≤0.0001). Lysine catabolism in cool season cereals produces piperidinecarboxylate [86], which is caused by the up regulation of the lysine-ketoglutarate reductase-saccharopine dehydrogenase (LKR/SDH) pathway during stress [87]. Lysine, like arginine, has been shown to protect protoplasts from lysis due to osmotic shock [73]. Galston et al. [73] speculated that the ultimate role of lysine in protecting against lysis by osmotic stress might be the production of polyamines that require lysine and arginine for their synthesis. It should be mentioned that wheat COR39, a cold induced protein, also has lysine rich unit, KR [88].

Of the aromatic amino acids, phenylalanine and tryptophan, only tryptophan displayed a change in concentration in crowns after freezing as compared to control crowns (Table 1). Tryptophan significantly increased in crowns at 3 d and 7 d (*P*≤0.05) and at 14 d (*P*≤0.01) (Table 1). The coefficient of determination of changes in tryptophan levels with DAF was highly significant, although the slope of the increase was slight. The study of temperature shock with *Arabidopsis thaliana* indicated that both phenylalanine and tryptophan are increased by cold shock but not with heat shock [63]. In contrast, the study with *Arabidopsis lyrata* ssp. *petraea* populations from different latitudes [52], found that under cold acclimating conditions there were no differences in tryptophan in plants from any latitude and only plants from the middle latitude, Sweden, had a significant increase (*P*≤0.05) in phenylalanine.

Many non-protein amino acids are involved in stress responses [89]; however, in this study only two responded significantly to freezing. 5-Oxoproline (i.e. pyroglutamate) in frozen crowns increased very significantly compared to levels in control crowns from 3 d through 14 d and the coefficients of determination with DAF were very significant and fairly strong (Table 1). In *Arabidopsis,* 5-oxoproline increases due to cold shock but not heat shock [63]. In contrast, in *Arabidopsis lyrata* ssp. *petraea* populations from different latitudes [52], 5-oxoproline significantly decreased in plants from Iceland (highest latitude) and increased in those from Wales (lowest latitude). Additionally, in a study of *Picea* populations undergoing cold acclimation at different latitudes 5-oxoproline was also shown to increase in the most southern-grown populations [90]. 5-Oxoproline has also been shown to be a significant contributor to the prediction of freezing tolerance in Arabidopsis populations segregating for freezing tolerance [53]. 5-Oxoproline is a major product of glutathione degradation in *Arabidopsis* [91].

The heterocyclic non-protein amino acid piperidinecarboxylate (i. e. pipecolic acid) increased significantly in frozen crowns at 3 and 14 d compared to levels in unfrozen controls and the coefficients of determination of these increases with DAF were modest although highly significant (Table 1). Although highly significant, the slope of the increase was relatively low in comparison to that for 5-oxoproline and proline. Piperidinecarboxylate appears to be a primary product of lysine catabolism in cool season cereals [86]. In halophyte plants such as seaside arrowgrass, piperidinecarboxylate accumulates along with proline when subjected to salt stress [92]. Also, osmotic shock has been found to increase piperidinecarboxylate in rapeseed leave tissues [87]. The degradation of lysine to piperidinecarboxylate during stress appears to be primarily due to the up-regulation of the LKR/SDH pathway [87]. It has been speculated that lysine catabolism via the LKR/SDH pathway in plants is important in reaction to stress in that glutamate, another catabolite along with piperidinecarboxylate, is necessary for the production of proline, arginine, and GABA, all of which are important in resistance to stress [93].

#### Sugars

Sucrose concentrations decreased significantly in the frozen crowns relative to control crowns at 0, 3 and 14 d and the coefficients of determination, and thus the correlation coefficients, were highly significant with DAF (Table 2; Figure S2). The negative slope of the sucrose decline after freezing was steeper than for any other metabolite with the exception of malate (Tables 1–3). In the study with *Arabidopsis lyrata* ssp. *petraea* populations from differing latitudes [52], sucrose concentrations were not significantly different in plants from Iceland (highest latitude) under cold acclimating conditions, but were significantly higher in plants from Sweden and Wales (lower latitudes). In fact, ecotypes from the lowest latitude (Wales) and mildest winters were the only ones that showed significant increases in sucrose and several other sugars (glucose, mannose, fructose); whereas, the ecotypes from the highest latitude and coldest winters (Iceland) had no significant increase in any sugar. In contrast, the study of temperature shock with *Arabidopsis thaliana* indicated that sucrose concentrations are increased by cold shock and heat shock [63].

Fructose concentrations increased significantly in the frozen crowns relative to control crowns at 0 to 3 d and the coefficients of determination, and thus the correlation coefficients, were significantly associated with DAF (Table 2; Figure S2). Yet the slope was negative because fructose concentration decreased in both frozen and non-frozen tissues 14 days after freezing. In oat crowns that had been freeze-acclimated at -3°C, fructose and fructan exohydrolase activity were significantly higher than in non-acclimated and cold-acclimated crowns [94]. The data presented here, suggest that after freezing either fructose is being depleted due to catabolism or that fructan is being synthesized, causing a reduction in the fructose pool. Fructan is a polymer of fructose with differing linkages and is a major storage carbohydrate in cool season grasses, such as oat [8].

The coefficients of determination for changes in turanose levels with DAF and significant and the slope in increase in turanose levels over time was moderate. Turanose and palatinose induce extracellular invertase (e.g. Lin6) mRNA levels in some species [95] and could be involved in sugar signaling in the freeze recovering tillers.

Of the other sugars and sugar related compounds, according to slope statistics, concentrations of most either had significant changes while decreasing slightly (gulose, maltose, melobiose, myo-inisitol, xylulose), increasing slightly (arabinose, ribitol, sorbitol, turanose) or showed no significant change in concentrations (arabinoate, galactose, glucose, glucuronate, 2-keto-gluconate, 4-keto-glucose, mannose, palitinose). These data indicate that little perturbation occurred with freezing and recovery except with levels of sucrose and fructose. This is similar to that reported by Davey et al. [52] with cold acclimation of ecotypes of *Arabidopsis lyrata* ssp. *petraea* that are the most cold hardy.

#### TCA cycle

Citrate and malate decreased significantly in frozen crowns compared to nonfrozen controls (Table 3; Figure S3). Decreases in citrate were significant (*P*≤0.01) at 7 d and 14 DAF and the coefficients of determination with DAF were highly significant (*P*≤0.0001) (*r* ^2^ = 0.76 and 0.78, respectively) (Table 3). Also, the negative slope of the decrease in citrate levels was quite high. Malate in frozen crowns also decreased significantly at 3 to14 DAF (*P*≤0.01) and the coefficients of determination for changes in malate levels versus DAF were highly significant (*P*≤0.0001) (Table 3). The negative slope of malate was by far the highest (negative or positive) slope found for any metabolite in this study (Tables 1–3). All other TCA cycle intermediates decreased to a lesser degree (Table 3). This is in contrast to studies on either temperature stressed or cold acclimated plants which found increases in most TCA cycle intermediates [e.g. 52; 63]. The Kaplan et al. [63] study did show a decrease in isocitrate with cold shock, although all other TCA cycle intermediates increased. The data presented here could suggest that after freezing the TCA cycle is either depleting substrates rapidly or that little substrate (e.g. pyruvate) is being fed into the TCA cycle. A study with very cold hardy and non-cold hardy cultivars of alfalfa demonstrated that during cold acclimation the root respiration Arrhenius E_a_ is lower allowing for a more rapid respiration rate at low temperature, and an overall rate of respiration that is higher for the cold hardy cultivar than for the cultivar with low hardiness [76]. This suggests that during cold acclimation mitochondrial respiration would be much higher in the more cold hardy cultivar and that a rapid turnover of TCA intermediates occurs at low temperature during cold acclimation. Without a direct measure of respiration in tissues which have undergone freezing it is difficult to determine the reason for the lower levels of TCA cycle intermediates as found in this study.

#### Lipid metabolism

In general, most changes in levels of metabolites involved in lipid metabolism in crowns that were frozen were of low enough magnitude to be nonsignificant (Table 3; Figure S3). Glycerol significantly decreased in frozen crowns as compared to nonfrozen controls at day 7 and the coefficients of determination with DAF were highly significant (*P*≤0.0001) (Table 3). The slope of the decrease was only moderate although also highly significant (*P*≤0.0001). Glycerol did significantly increase in frozen crowns relative to controls at day 0 and may be similar to the *Arabidopsis* response to temperature stress documented by Kaplan et al. [63], where increases in glycerol were found with both cold and heat shock. Stearic acid decreased in frozen tissues in relation to non-frozen tissues only at 1 d. Although the coefficients of determination of changes in stearic acid levels in frozen tissues were significant with DAF, the slope of decrease was slight.

#### Other Metabolites

As compared to nonfrozen crowns, phosphoric acid concentrations were significantly lower on 1, 7 and 14 d in crowns that had been frozen, although phosphoric acid increased in both frozen and non-frozen crowns (Table 3; Figure S3). The coefficients of determination for changes in phosphoric acid were highly significant for DAF (*P*≤0.0001), with a highly significantly slope for the regression line with DAF (Table 3). Kaplan et al. [63] found that phosphoric acid increased with cold shock and decreased with heat shock in *Arabidopsis*. Lactate decreased significantly at 3 d and 14 d in frozen crowns as compared to nonfrozen crowns, although lactate decreased in both frozen and nonfrozen crowns (Table 3). Also, the correlations of determination of changes in lactate levels were significant (*P*≤0.001) with days after freezing. Cold shock was found to decrease lactate concentrations in *Arabidopsis* while heat shock had no affect [63]. Butanoate, a product of glutamate catabolism, levels increased significantly (*P*≤0.05–0.01) at 3 d, 7 d and 14 d and the coefficients of determination were highly significant (*P*≤0.001) with days after freezing, although the increase was not great (Table 3). Rythronic acid (i.e. tetronic acid), a plant antimicrobial compound [96] increased in frozen crowns significantly (*P*≤0.05) as compared to nonfrozen control crowns at 14 d and coefficients of determination were very significantly (*P*≤0.0001) associated with days after freezing, although the slope of increase was slight (Table 3). Other metabolites listed in Table 3 either did not have significant changes or only minor changes in concentration over the course of the experiment.

#### Safranin staining and metabolite changes after freezing

We have previously reported the development of a spherical mass of safranin staining tissue over the two weeks following freezing of oat crowns [31]. Those tissues were produced using the same protocol as used in this metabolomics study. Hence, we have correlated the formation of this safranin staining tissue with changes in metabolites over the same period to determine if there is any relationship between the two. Concomitant with this increase in safranin staining were highly significant positive correlations with a number amino acids and related compounds (Table 1). These correlations were especially significant (*r* ^2^≥0.75 0.0001, *P* ≥0.0001) for arginine, asparagine, histidine, lysine, and tryptophan. In contrast, sugars and related compounds, which generally decreased over this period, either did not correlate or did not correlate as well (Table 2). Of the other metabolites detected, two TCA intermediates, citrate and malate, were highly negatively correlated (*r* ^2^≥0.75 0.0001, *P* ≥0.0001) with safranin staining of crown tissue. This could suggest either a more rapid turnover of TCA intermediates or less substrate entering the TCA cycle associated with safranin staining.

#### Principal component analysis

**Figure 2:**
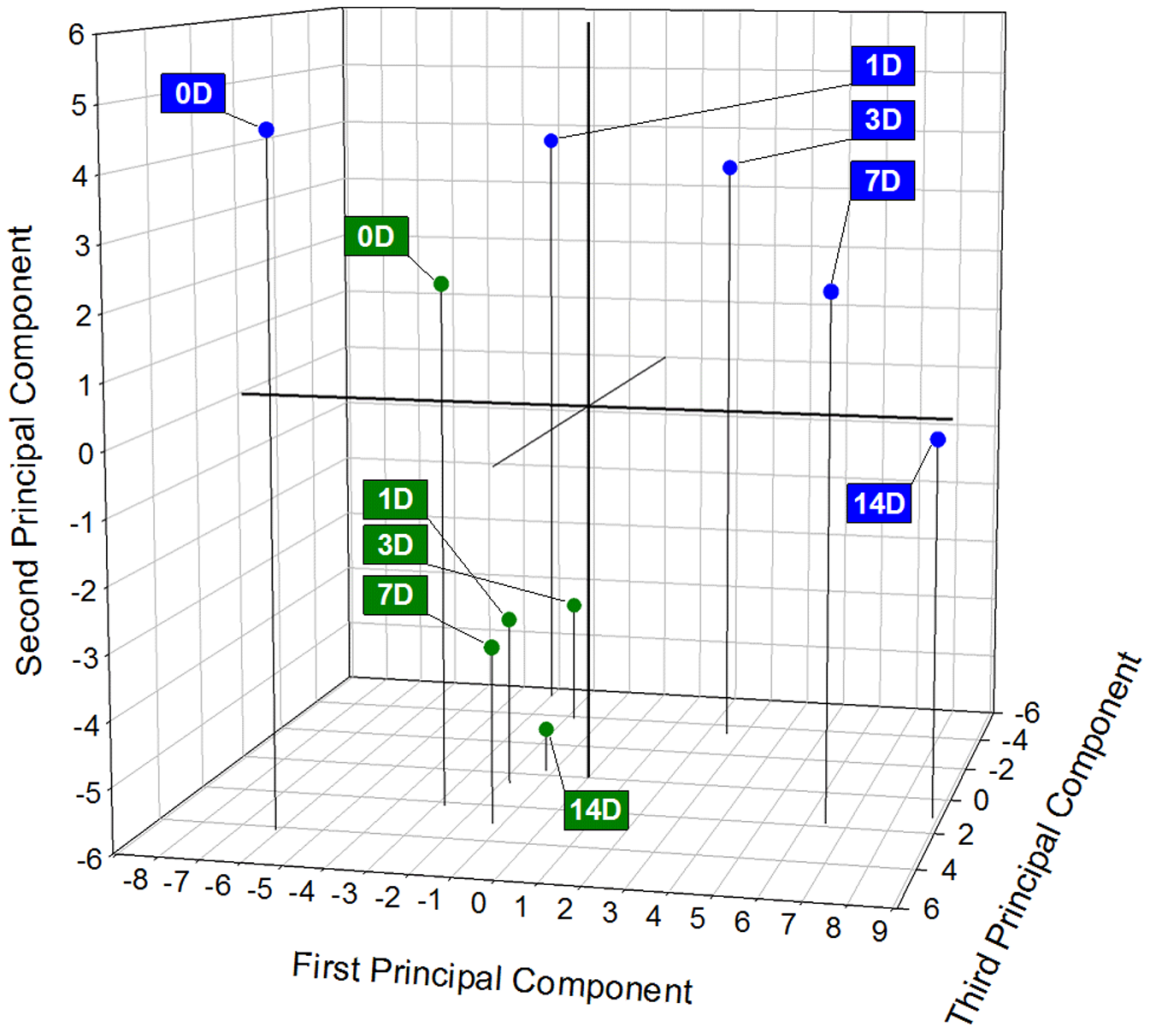
Loading plot with superimposed scores for the first three vectors from principal component analysis of GC-MS detected metabolites from frozen (blue) and control (green) crowns of cold acclimated oats during 0 to 14 days of growth after imposition of freezing stress.

Frozen crowns were separated from unfrozen crowns by Principal Component Analysis (PCA) which captured 83% of the variation between the samples in the first 5 PCs (Table 4). At 0 d both frozen and control crowns were separated from crowns analyzed at all later days in the time course by PC1, which accounted for 32% of the variation, where 0 d crowns were more negative than crowns from later days (Figure. 2). The metabolites with the most positive loading values for the first vector starting with the most positive were: butanoate, 5-oxoproline, valine, piperidinecarboxylate, leucine, isoleucine, arginine and threonine. The metabolites with the most negative loading values for this vector, starting with the most negative were: citrate, cinnamic acid, isocitrate, sucrose and glycerol. When returned to optimal growth conditions, control crowns were separated based on length of exposure to optimal conditions by PC2, where 0 d control crowns were positive and control crowns from all later days were negative. Frozen crowns from 0 d were separated from frozen crowns from all other days primarily by PC1 although over time the crowns that had been frozen became more negative on PC2, which further separated them from the 0 d frozen crowns. The second vector accounted for 19% of the variation between the samples and the metabolites with the most positive loading values for the second vector were, starting with the most positive, 4-keto-glucose, glycine, erythrose, GABA, and serine. The metabolites with the most negative loading values for the second vector were, starting with the most negative, aspartate, phosphoric acid, malate and sucrose. The third vector accounted for 13% of the variation between the samples for a cumulative value of 64%. The metabolites with the most positive loading values for the third vector were, starting with the most positive, bis-propyl-phosphoric acid, melibiose, gulose and glucuronate and the metabolites with the most negative loading values were mannose, erythritol, xylulose and fumarate. It is interesting to note that frozen crowns at 14 d had shifted metabolically which resulted in a more negative position on PC2 (Figure. 2). It is tempting to speculate that this shift towards the cluster of control crowns on PC2 represents recovery of the crowns from freezing stress.

**Table 4:** Results from principal component analysis of GC/MS metabolomic data of frozen and Nonfrozen oat crowns during days 0 to 14 of regrowth.

Principal Component		Variation Explained	
	Eigenvalue	Proportion	Cumulative
			(%)
1	173	0.32	32.1
2	102	0.19	51.0
3	70	0.13	64.1
4	64	0.12	75.9
5	38	0.07	83.1

## Conclusion

Winter hardiness is arguably the most complex issue related to successful cultivation of winter cereals. Numerous reports on cold and freeze acclimation have described various freezing survival mechanisms. One aspect of winter hardiness that has received little attention is the period of regrowth following freezing when repair mechanisms could significantly impact how or if a plant survives freezing. While it is doubtful that one or two simply inherited mechanisms will explain this important aspect of freezing survival, a better understanding of this post-freeze period could provide a more comprehensive understanding of overall winter hardiness. In this study, we demonstrate that there are distinct metabolic changes that distinguish frozen plants, upon return to optimal growth temperatures, from plants that have not been freeze stressed. We have shown that some alterations in amino acid pools after freezing were similar to those observed in cold acclimation whereas most changes in sugar pools after freezing were not. These similarities and differences suggest that there are common as well as unique genetic mechanisms between these two environmental conditions that are crucial to the winter survival of plants.

## Supporting Information

Figure S1 Changes in amino acids and related metabolites in frozen and nonfrozen oat crowns during days 0 to 14 after freezing.
(PDF)

Figure S2 Changes in sugar related metabolites in frozen and nonfrozen oat crowns during days 0 to 14 after freezing.
(PDF)

Figure S3 Changes in additional metabolites in frozen and nonfrozen oat crowns during days 0 to 14 after freezing.
(PDF)

Table S1 Loading values for the first five principal components.
(XLSX)
