# Supplementary figures and images for: Metabolic Changes in Avena sativa Crowns Recovering from Freezing
Source: PLoS One. 2014 Mar 27;9(3):e93085. doi: 10.1371/journal.pone.0093085 (PMC3968094; doi:10.1371/journal.pone.0093085)

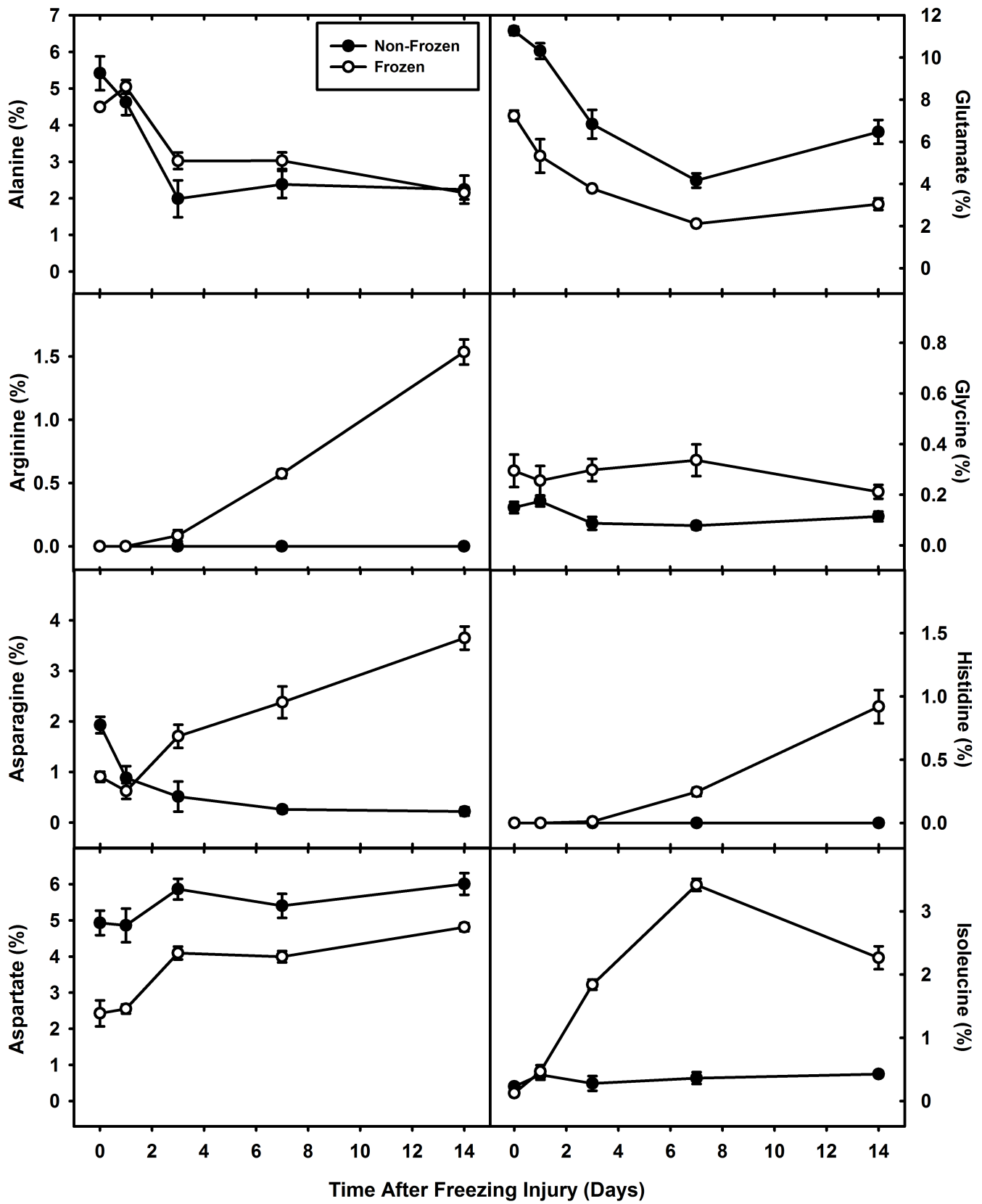

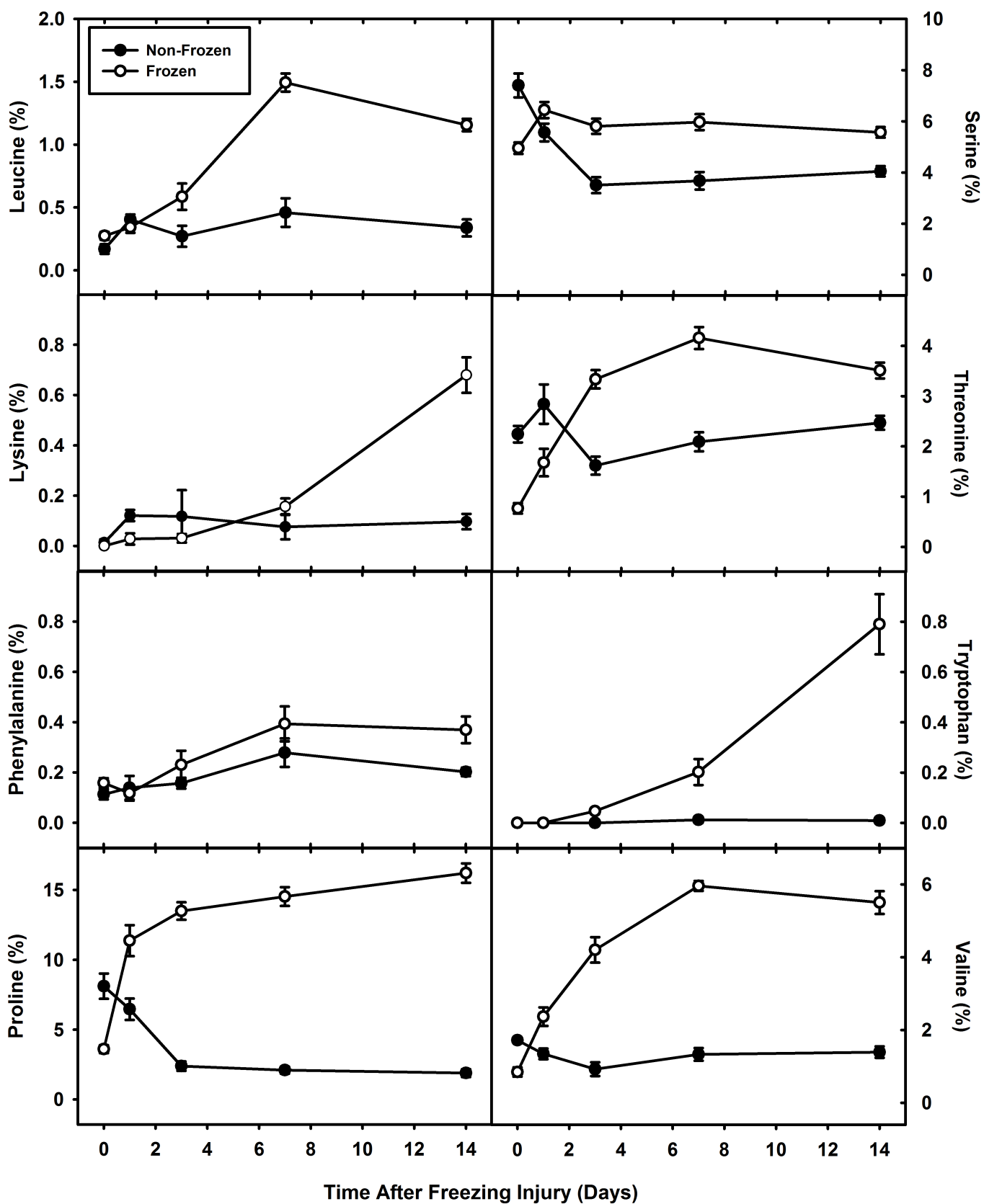

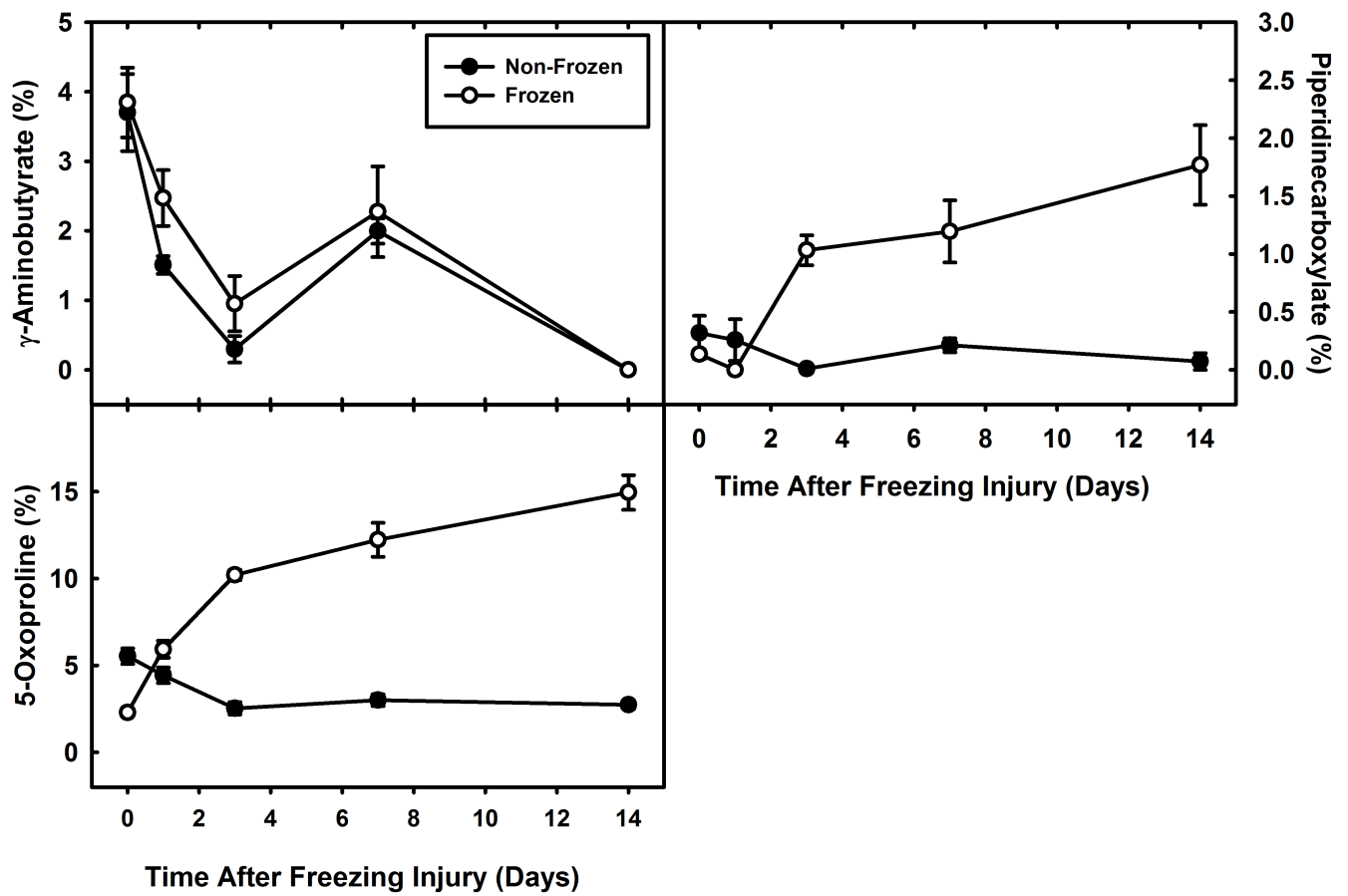

Supplement: Figure S1 — Changes in amino acids and related metabolites in frozen and nonfrozen oat crowns during days 0 to 14 after freezing. (PDF) [file pone.0093085.s001.pdf]

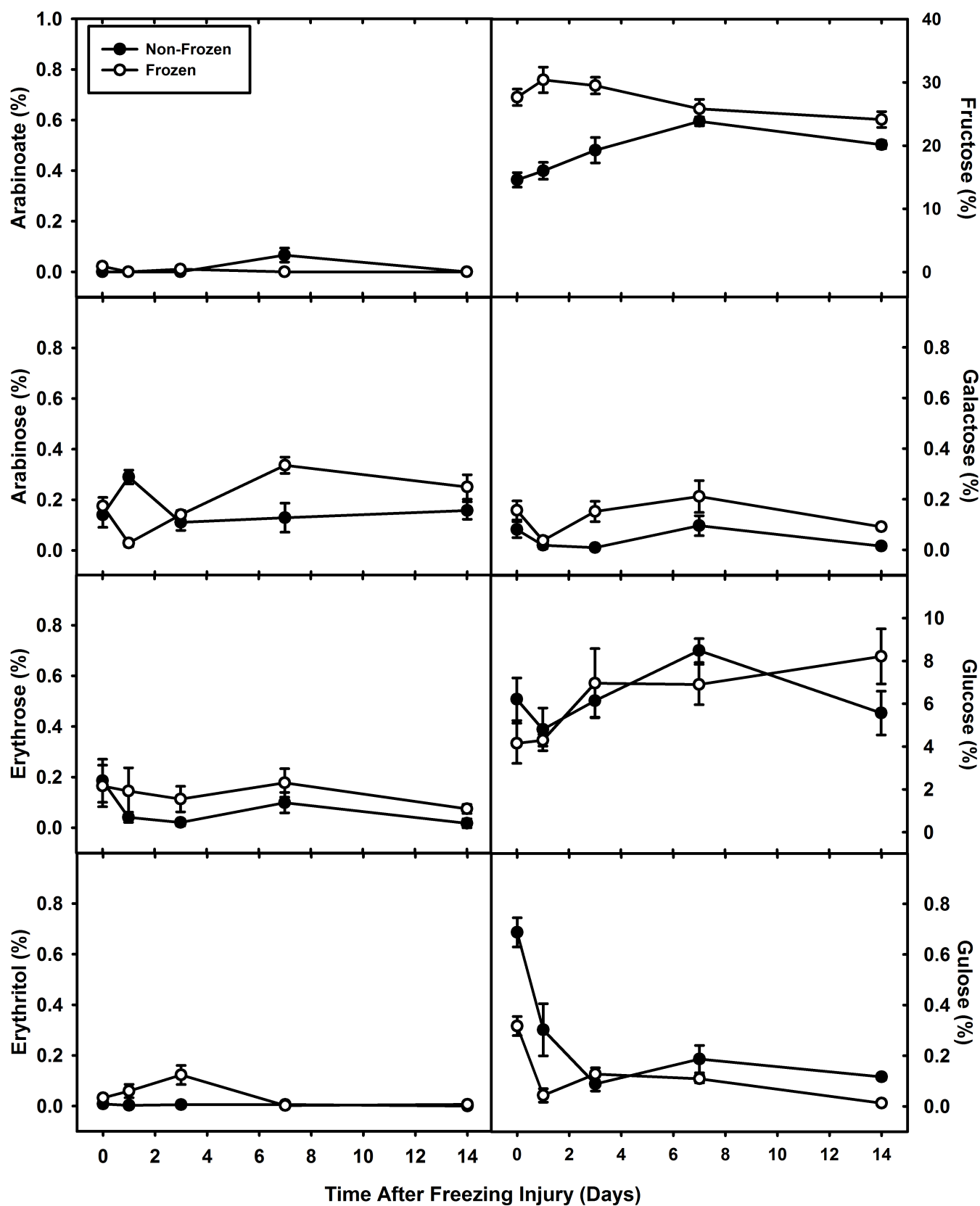

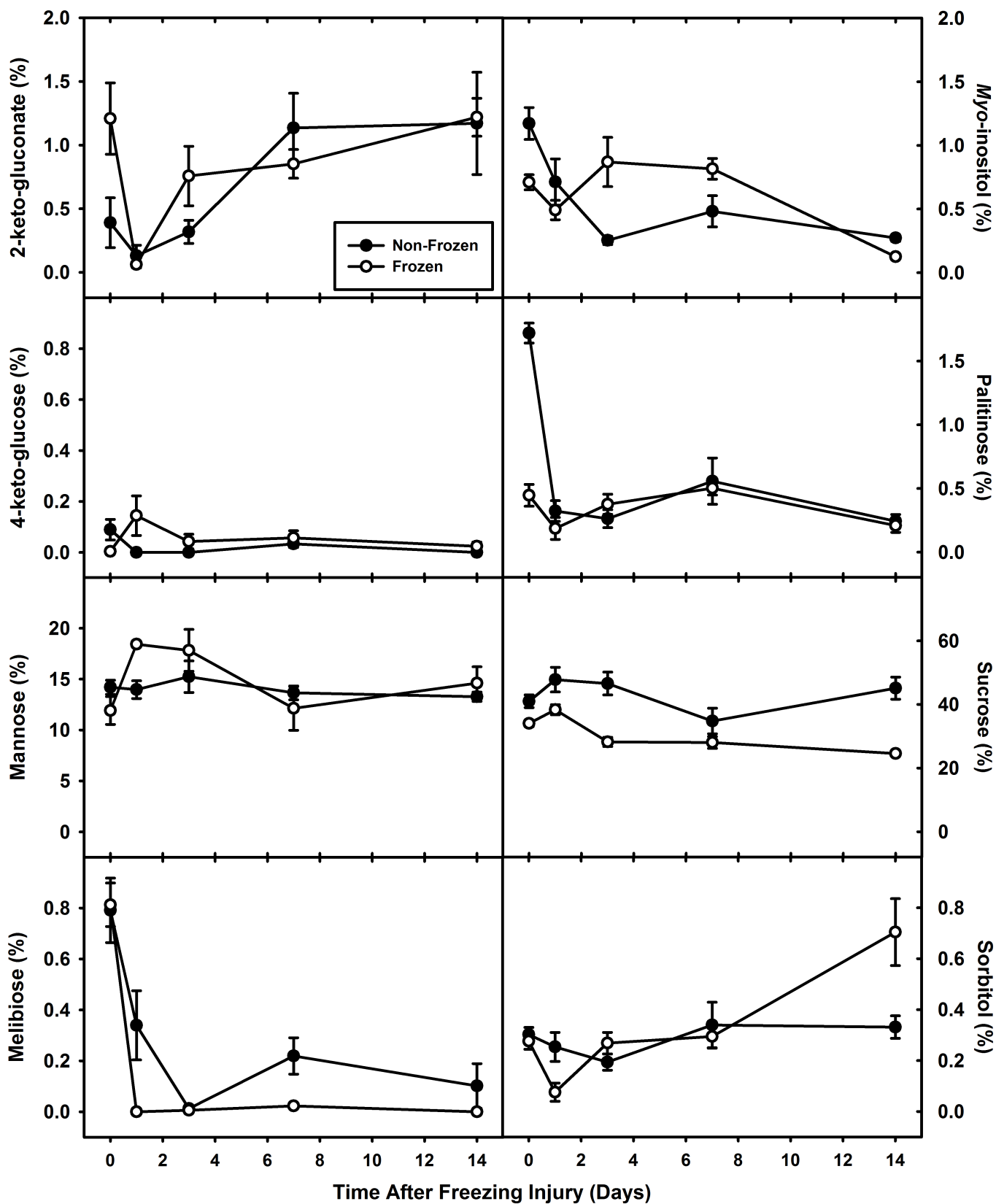

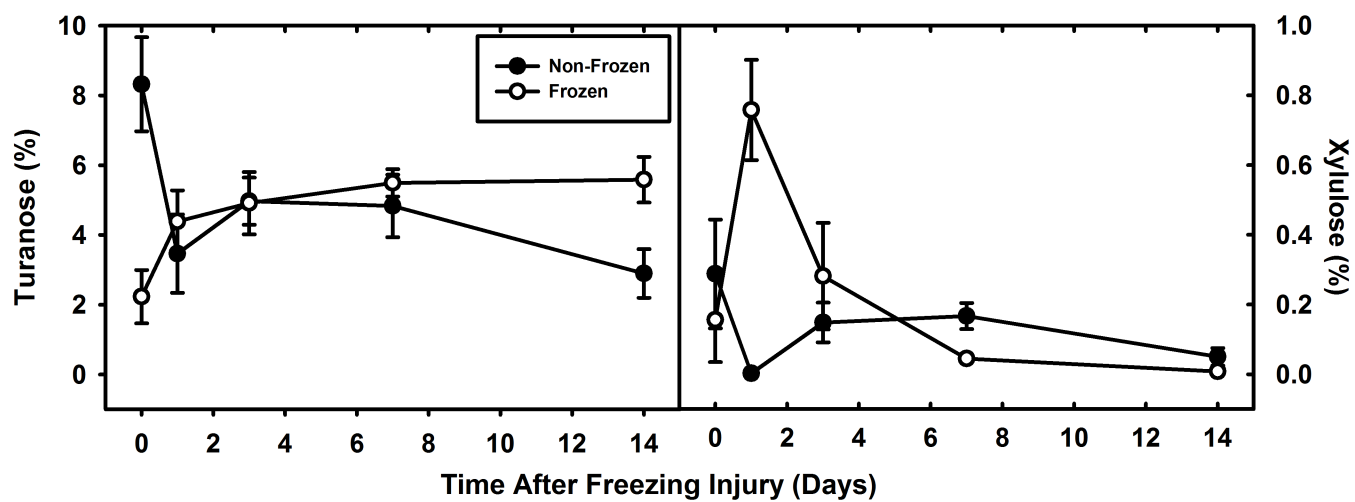

Supplement: Figure S2 — Changes in sugar related metabolites in frozen and nonfrozen oat crowns during days 0 to 14 after freezing. (PDF) [file pone.0093085.s002.pdf]

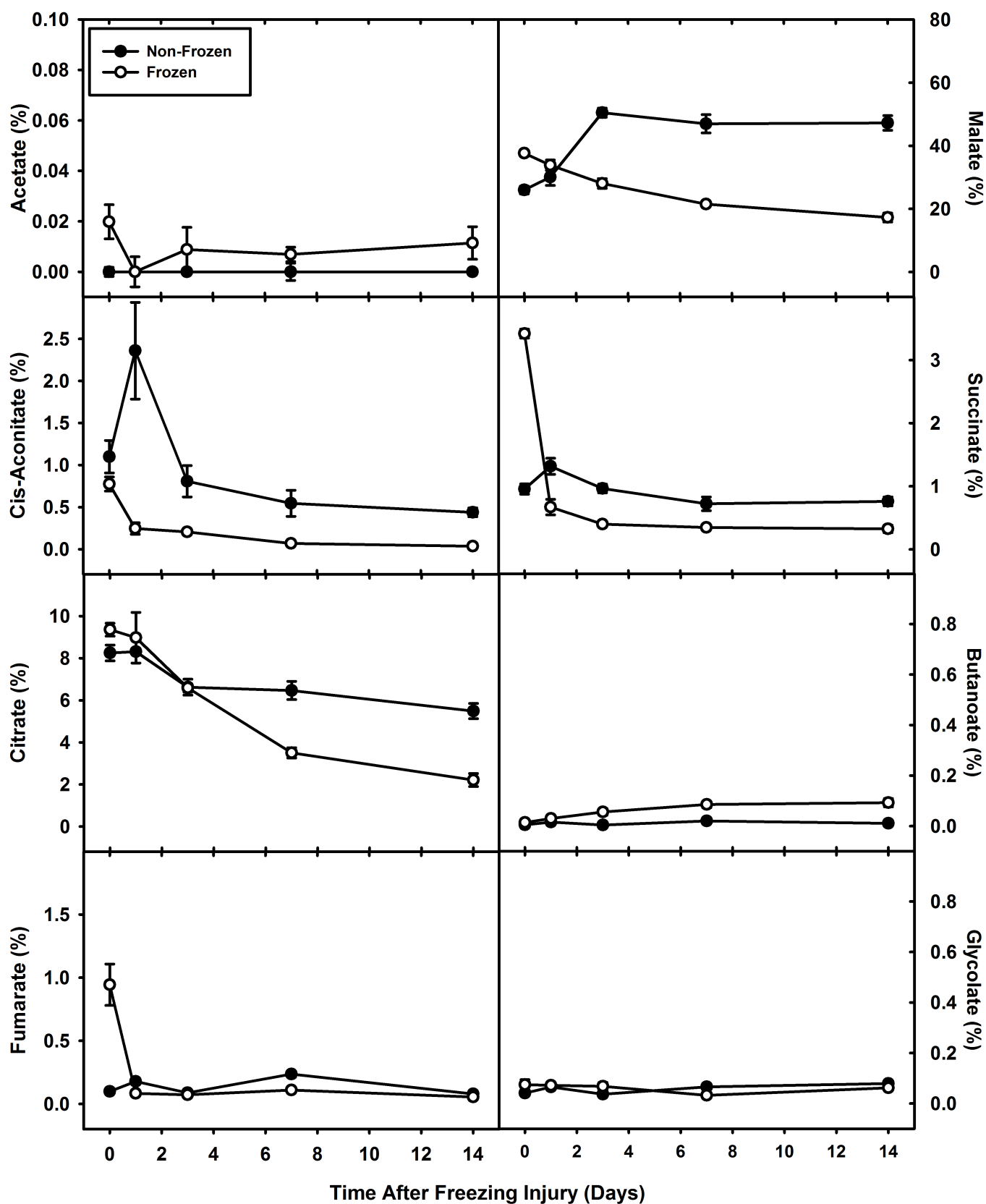

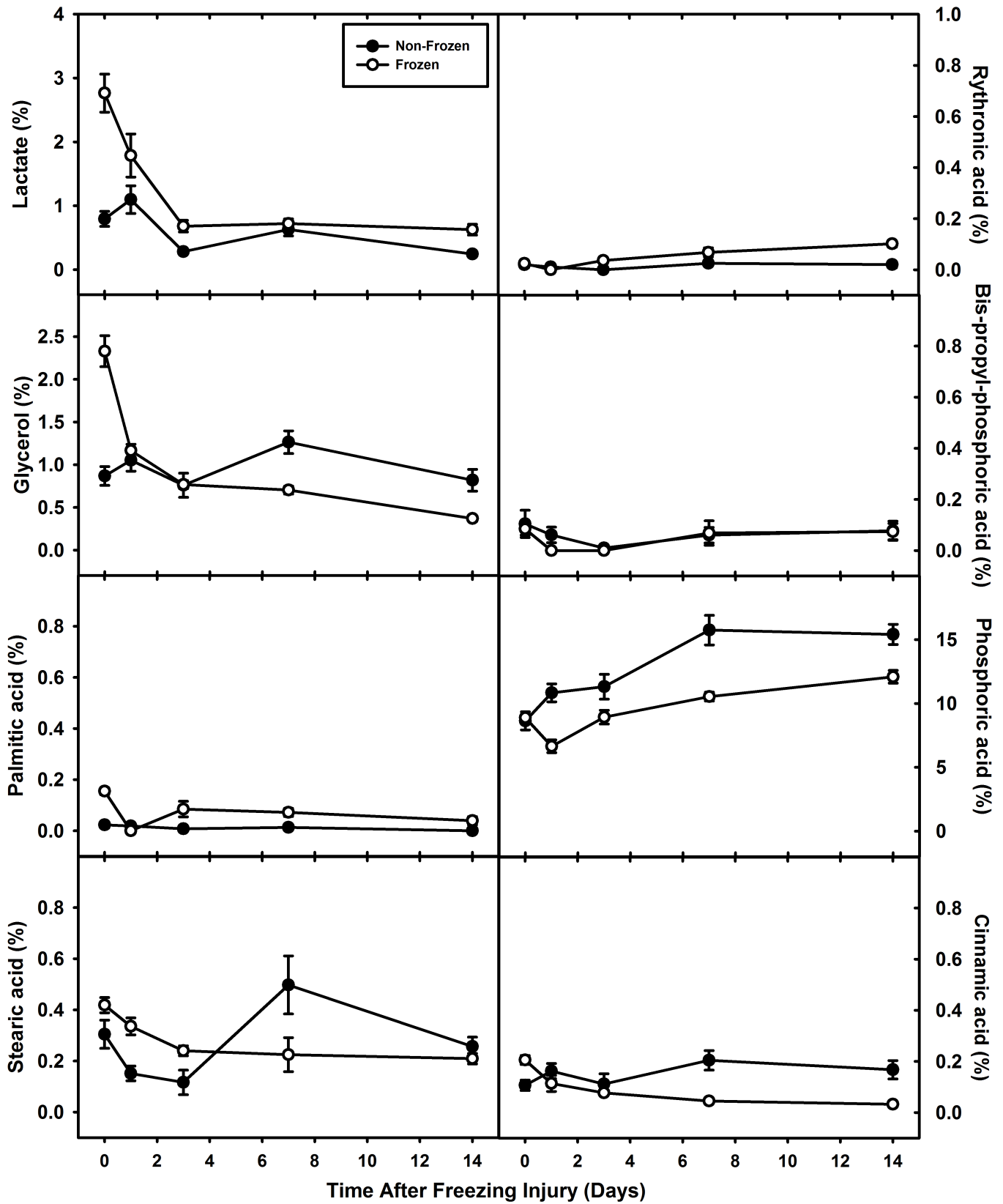

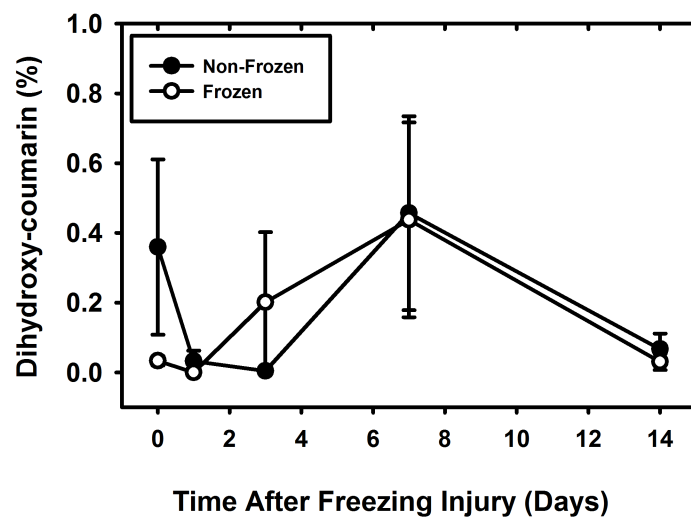

Supplement: Figure S3 — Changes in additional metabolites in frozen and nonfrozen oat crowns during days 0 to 14 after freezing. (PDF) [file pone.0093085.s003.pdf]
